# From Environmental Persistence to Host Adaptation: Differential Curli Regulation in *Salmonella enterica* Serovars Typhimurium and Typhi

**DOI:** 10.3390/microorganisms14061289

**Published:** 2026-06-07

**Authors:** Camille Ou, Karine Dufresne, Charles M. Dozois, France Daigle

**Affiliations:** 1 Département de Microbiologie, Infectiologie et Immunologie, Université de Montréal, 2900 Boulevard Édouard-Montpetit, Montreal, QC H3T 1J4, Canada; camille.ou@umontreal.ca; 2 CRIPA, Centre de Recherche en Infectiologie Porcine et Avicole, Faculté de Médecine Vétérinaire, 3200 Sicotte, St-Hyacinthe, QC J2S 2M2, Canada; charles.dozois@inrs.ca; 3 Département des Sciences Biologiques, Université du Québec à Montréal, 405 Rue Sainte-Catherine Est, Montreal, QC H2L 2C4, Canada; dufresne.karine.3@uqam.ca; 4 Centre Armand-Frappier Santé Biotechnologie, Institut Nationale de la Recherche Scientifique (INRS), 531 Boulevard des Prairies, Laval, QC H7V 1B7, Canada; 5 Institut Courtois d’Innovation Biomédicale, Université de Montréal, Montreal, QC H3T 1J4, Canada

**Keywords:** curli fimbriae, regulation, *Salmonella* Typhimurium, *Salmonella* Typhi

## Abstract

*Salmonella enterica* comprises numerous serovars with distinct host ranges and disease outcomes. Among them, *Salmonella enterica* serovar Typhimurium (*S*. Typhimurium) is a leading cause of gastroenteritis worldwide. Its ability to persist both in the environment and within the host gastrointestinal tract is largely attributed to biofilm formation. In contrast, the human-restricted pathogen *Salmonella enterica* serovar Typhi (*S*. Typhi) primarily forms biofilm in the gallbladder during chronic infection. These differences suggest that the two serovars are exposed and respond to distinct environmental cues. Curli fimbriae are key components of the biofilm matrix, contributing to initial surface adhesion and structural stability. In this review, we examine the regulation of curli fimbriae (*csg* operons) in *S*. Typhimurium, incorporating recent advances in the field, and compare these mechanisms with new insights concerning regulation in *S*. Typhi. Comparative analyses highlight significant differences in *csg* expression and regulatory pathways between the two serovars. All two-component systems known to influence curli expression carry mutations in active protein domains in *S.* Typhi. This is also true for diguanylate cyclases and phosphodiesterases, with some exhibiting important modifications in *S.* Typhi, including truncation and insertion. Such polymorphisms could contribute to variation in the curli regulatory pathway and may reflect broader mechanisms of host adaptation in *S.* Typhi. Understanding this regulatory divergence is essential for elucidating host specificity and the distinct pathogenic strategies of *S*. Typhi related to biofilm formation.

## 1. Introduction

*Salmonella* are enterobacteria sub-classified into more than 2500 serovars dependent on the O-antigen (O), flagella (H), and the Vi capsule [1,2]. Non-typhoidal *Salmonella* (NTS), such as *Salmonella enterica* serovar Typhimurium (*S.* Typhimurium), is among the leading causes of acute diarrheal disease worldwide [3,4]. Typhoidal *Salmonella* (TS), such as *Salmonella enterica* serovar Typhi (*S.* Typhi), is the causative agent of typhoid fever, a systemic infection restricted to humans. Treatment of *S.* Typhi infections has become increasingly complex with the emergence of multidrug-resistant (MDR) and extensively drug-resistant (XDR) strains. Following acute infection with *S.* Typhi, approximately 3–5% of individuals become chronic carriers and can shed the pathogen for more than a year [5] to several years post-infection in some cases [6]. Chronic carriage is associated with biofilm formation on gallstones in the gallbladder, currently considered the only known reservoir of *S.* Typhi, promoting long-term bacterial persistence [7]. While regulation of biofilm in *S.* Typhi is not well understood, biofilm formation by *S.* Typhimurium has been extensively characterized [8,9,10]. Unlike *S.* Typhi, *S.* Typhimurium exhibits a broad host range, infecting humans, birds, reptiles and other animals [11]. It also survives outside the host in diverse environments, including soil, water, plants, and abiotic surfaces, largely through biofilm formation [12,13]. The biofilm extracellular matrix of *S.* Typhimurium is composed of several key components, including the biofilm-associated protein BapA [14], extracellular DNA (eDNA) [15], exopolysaccharides such as cellulose [16,17], the O-antigen capsule [18], and curli fimbriae [8,19,20]. Curli are extracellular amyloid fibers that are essential for adhesion and can account for up to 85% of the biofilm matrix in some species, like *E. coli* [21]. Purified curli fibers are highly resistant and not easily depolymerized by either high temperatures or detergent treatment, providing stability and environmental protection [22,23]. Curli production is tightly regulated by environmental cues [24]. Under laboratory conditions, expression is induced in nutrient-limiting or starvation conditions, such as growth in LB medium without salt or in tryptone, and at temperatures below 30 °C [24,25]. Oxygen availability also plays an important role in curli regulation [26,27]. In the presence of Congo red agar, *S.* Typhimurium produces a red, dry and rough colony biofilm phenotype (rdar), which is associated with cellulose and curli production [17]. Under the same growth conditions, *S.* Typhi presents a smooth and white (saw) phenotype associated with the absence of curli and cellulose expression [28].

While curli are primarily associated with environmental persistence and biofilm formation outside the host, some studies have shown that these fimbriae can adhere to several host cells, suggesting an important role in the initial stage of the infection [29,30]; they are also expressed during infection in animal models [31,32]. Curli interact with plasma proteins, such as fibronectin, laminin, plasminogen, and MHC class I molecules, and can trigger the production of pro-inflammatory cytokines, such as IL-6, IL-8, TNF-α, and nitric oxide [31,33]. These interactions contribute to local inflammation and can lead to intestinal persistence following biofilm formation [34].

**Figure 1:**
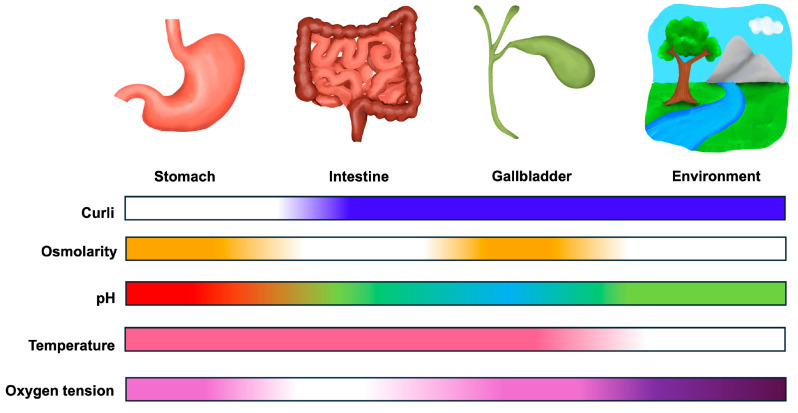
Schematic representation of environmental conditions encountered by *S.* Typhimurium and their impact on curli expression. Curli are expressed for environmental persistence and are potentially also expressed in the intestine and gallbladder of the host (dark blue). The stomach and the gallbladder are high in osmolarity (orange), while the intestine and the environment are neutral (white). The pH of the stomach is acidic (red), while the gallbladder is alkaline (blue). The intestine and the environment are neutral pH (green). The temperature inside the human host is 37 °C (pink). The temperature of the environment is under 30 °C (white). Oxygen tension is highest in the environment (dark purple). Inside the stomach, oxygen tension is reduced. It continues to drop through the intestine (white). Internal organs such as the gallbladder are oxygenated through blood circulation.

During the infection, *Salmonella* is exposed to multiple stress signals within the host (Figure 1). One of the first cues is the increase in temperature upon entering the human body. As bacteria pass through the stomach, they must withstand strong gastric acidity, which induces the acid-tolerant response (ATR), as well as elevated osmolality and reduced oxygen availability [35,36,37]. As *Salmonella* transits into the small intestine, environmental conditions shift toward a near-neutral pH, low oxygen tension and an isosmotic environment (300 mOsm/kg), corresponding to the internal osmolality of *Salmonella* and that of human plasma [37,38,39,40]. Curli production is favored under neutral-to-alkaline conditions, whereas acidic environments strongly repress its expression [24]. This suggests that gastric acidity may suppress curli production until *Salmonella* reaches the ileum and colon, where the pH is closer to neutrality. In addition to pH and osmolarity, metabolite availability modulates curli expression. High concentrations of nitrate [41], glucose [42] or iron [25,42] repress curli production, and these metabolites are typically present at lower levels in the ileum and colon. In contrast, *S.* Typhi does not induce strong local intestinal inflammation during its transit in the intestinal tract [43]. Bacteria break the intestinal barrier and enter phagocytes, leading to systemic dissemination. The liver, the spleen, the bone marrow and the gallbladder are colonized with *S.* Typhi. Rather than persisting in the intestine, *S.* Typhi preferentially forms biofilm in the gallbladder, which can contribute to chronic carriage [44,45,46]. This distinct pathogenic strategy implies that additional environmental or host-derived cues may be involved in curli regulation in this serovar.

Curli regulation has been extensively studied in *E. coli*, but much less is known in *Salmonella*. While the two species share many regulatory features, findings from *E. coli* studies are often inadequately extrapolated to *Salmonella* without direct experimental validation. Several regulators have been shown to regulate curli expression in *E. coli*, without supporting evidence in *Salmonella*. In addition, regulatory pathways are not fully conserved across *Salmonella* serovars. For instance, *S.* Typhimurium and *S.* Typhi do not follow identical regulatory schemes. Although sharing 89% of their genome, *S.* Typhi displays a distinct genomic organization resulting from rRNA rearrangements, the presence of insertion elements such as *IS*200, as well as multiple deletions and differences in SPI and phage content [47,48,49,50,51,52]. Moreover, about 5% of the *S.* Typhi genome consists of pseudogenes, likely acquired during host adaptation [50]. In addition, multiple polymorphisms are distributed throughout their genomes, and their functional consequences, particularly on gene regulation, are still not well understood [27,53].

This review focuses on the regulation of curli fimbriae in *S.* Typhimurium as a reference framework to compare regulatory mechanisms in *S*. Typhi. By highlighting both conserved and divergent regulatory strategies, we aim to provide new insights into how genomic variations may reshape curli regulation and contribute to the virulence of *S.* Typhi.

## 2. Subunits: CsgB and CsgA

Curli fimbriae are composed of minor CsgB subunits and major CsgA subunits [54]. Both proteins share approximately 51% similarity and are composed of five repeated sequences (R1 to R5). The repeated sequences R1 to R4 are conserved in both proteins, while R5 presents more differences. The repeat sequences play distinct roles in curli polymerization [54,55]. CsgB and CsgA are secreted across the outer membrane through the CsgG-CsgF secretion channel [9,56,57]. At the cell surface, the R5 of CsgB interacts with CsgF, which anchors CsgB near the membrane. In contrast, R1 to R4 are amyloidogenic and show strong similarity to the five repeat sequences of CsgA [55]. This similarity suggests that R1 to R4 of CsgB contribute to the initiation of CsgA nucleation and self-polymerization at the bacterial surface. *S.* Typhi CsgB carries a missense mutation within the R2 repeat [27], and its impact on CsgB nucleation capacity remains unknown.

In CsgA, R1 and R5 contain the nucleation-responsive domains that allow seeding by CsgB [58]. Serine (Ser/S), glutamine (Gln/Q) and asparagine (Asn/N) residues are found across the CsgA sequence [59], forming a polar zipper motif that stabilizes the amyloid structure through multiple hydrogen bonds between side chains [60]. The high density of hydrogen bonds confers high resistance of curli filaments to temperature and chemical treatments, making them difficult to depolymerize [22,23]. Unlike many other extracellular appendages, curli assembly does not require a periplasmic chaperone. Filament elongation occurs at the tip of the filament rather than at the base, in a mechanism termed extracellular nucleation-precipitation (ENP). This process proceeds rapidly to prevent CsgA monomers from diffusing from the cell surface [61]. Although purified CsgA can self-polymerize in vitro in the absence of CsgB, this does not occur in vivo. Such tight spatial and temporal regulation is essential to prevent premature amyloid formation and cellular toxicity [55].

## 3. Anti-Fibrillation Protein CsgC

*csgC* is co-transcribed with *csgB* and *csgA* and is localized to the periplasm [62]. Given that CsgC lacks proteolytic activity, it is thought to function as a chaperone-like protein that prevents premature intracellular polymerization of CsgA. CsgC interacts with multiple regions of CsgA through electrostatic interactions, maintaining CsgA in a soluble, unfolded conformation, and thereby preventing intracellular amyloid formation and associated toxicity [63,64,65,66,67]. In vitro, CsgC has also been shown to inhibit polymerization of other amyloid-forming proteins, including *Pseudomonas* FapC and α-synuclein, which is associated with human neurodegenerative disease [63]. In *S.* Typhi, CsgC carries a missense mutation outside the predicted active domain, a modification that is unlikely to affect its function [27]. In *E. coli*, the periplasmic protein YedX, involved in purine metabolism [68], and protein YccT (renamed CsgI) [69] also inhibit intracellular polymerization of CsgA monomers. The periplasmic serine protease Prc, in collaboration with CsgC, degrades excess CsgA in the periplasm [70]. Similar gene products are also found in the genus *Salmonella* with a certain degree of conservation. Their role in *Salmonella* is yet to be determined.

## 4. Export Machinery: CsgG, CsgE and CsgF

All curli components are transported to the periplasm by the Sec pathway. In the periplasm, soluble CsgA is recognized by the pseudo-chaperone CsgE through its 22-amino-acid secretion signal (A22) [56,61,71]. If the CsgA signal peptide A22 is fused to other proteins of similar size, such as CpxP or PapD2, these cytoplasmic proteins can also be exported in the absence of CsgA [56,71]. Although CsgB and CsgF are exported extracellularly, they lack the A22 secretion signal, and their mechanism of secretion has not been identified yet. CsgE associates with the CsgG channel at the periplasmic side of the outer membrane, carrying CsgA along and trapping it within the channel cavity [57,62,71,72]. Acting as the “gatekeeper” of CsgG, CsgE prevents the export of unrelated proteins by plugging the periplasmic opening [22,61,71,72,73,74]. The curli export channel consists of a nonameric CsgG pore associated with nine CsgE subunits at the periplasmic side and nine CsgF subunits at the extracellular side [8,56,57,61,72]. Within the CsgG channel, CsgA interacts with the inner wall via a six-amino acid motif, forming hydrophobic contacts and hydrogen bonds [57]. CsgA then passes through two constriction points, one formed by CsgG and the other by CsgF, before secretion into the extracellular environment [57,72]. Each CsgF molecule interacts with two adjacent CsgG units, stabilizing the channel through its N-terminal binding to the inner wall of CsgG. The nonameric CsgF assembly forms a funnel-like structure [75]. On the extracellular side of the outer membrane, the C-terminus of CsgF interacts with the R5 of CsgB, linking CsgB to the pseudo-porin CsgG [57]. In *S.* Typhi, CsgE contains a missense mutation at position 36 related to self-association [27,76]. This mutation does not alter essential residues for interactions with either CsgA or CsgG [57], but protein interaction assays using *S.* Typhi CsgE are required to confirm this hypothesis.

## 5. The Intergenic Region of *csg*

**Figure 2:**
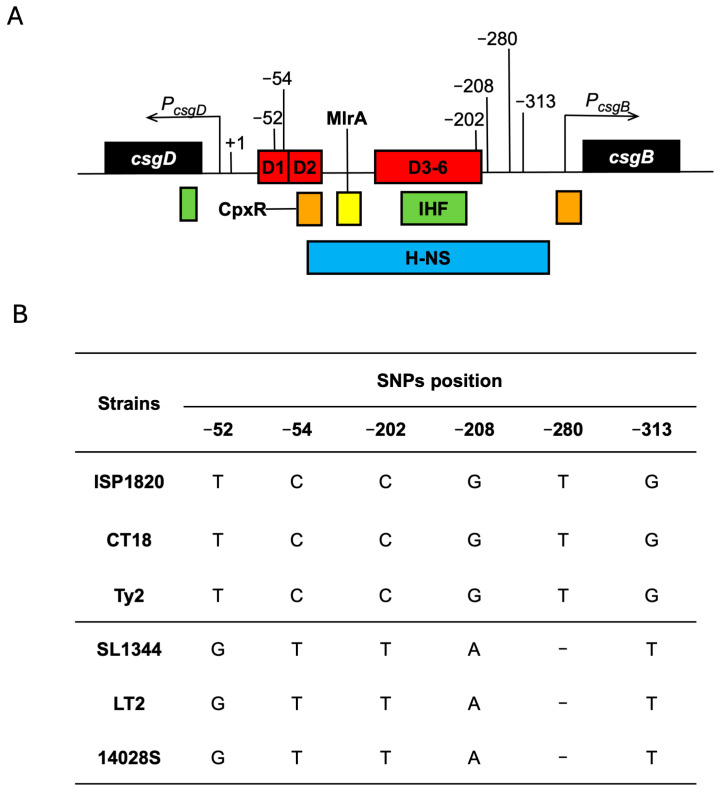
Schematic representation of the polymorphisms in the *csg* intergenic regions of *S*. Typhimurium and *S*. Typhi genomes. (**A**) Schematic representation of the native *S*. Typhi intergenic region containing *csgD* (in a black box) with its promoter (*P_csgD_*), *csgB* (in a black box) with its promoter (*P_csgB_*), and predicted OmpR binding sites (D1, D2 and D3-D6; red boxes). Predicted binding site of CpxR is in orange box, MlrA in yellow box, IHF in green box and H-NS in blue box. Positions of the 6 SNPs are indicated relative to the *csgD* transcription starting site (TSS). (**B**) A hundred strains of each serovar were compared, and all SNPs are conserved between them. The table compares these SNPs across 3 different *S*. Typhi (above) and *S*. Typhimurium (below) reference strains.

The two curli operons, *csgBAC* and *csgDEFG*, are divergently transcribed and separated by an intergenic region of 754 pb with untranslated regions (UTRs), which contain the *csgB* and *csgD* promoters. This large intergenic region exhibits intrinsic DNA curvature and reduced stability and flexibility compared to the rest of the genome [77]. Even a single nucleotide change within this region is sufficient to significantly alter curli expression [8,78], as it contains several overlapping binding sites for transcription factors and nucleoid-associated proteins, including OmpR, CpxR, MlrA, H-NS, IHF, RpoS, as well as regulatory small RNAs [26,79,80] (Figure 2A). Although additional regulators (e.g., RspA) have been reported to influence curli expression, their direct binding sites within this region have not yet been identified [79,81]. Collectively, these regulators integrate environmental and physiological cues through a complex regulatory network centered on the transcription factor CsgD [8,82]. The intergenic region of *S.* Typhi contains six SNPs scattered across distinct regulatory binding sites. These polymorphisms are highly conserved among *S*. Typhi strains (Figure 2B), suggesting potential functional relevance in shaping its specific regulatory landscape. SNPs in the intergenic region could alter the affinity of regulators to their binding sites. This will be discussed further in other sections.

## 6. The Internal Regulator of Curli Fimbriae: CsgD

CsgD is an orphan transcription factor belonging to the LuxR/UhpA/FixJ family [8,82]. Unlike many response regulators, CsgD is active in its unphosphorylated state and becomes inactivated upon phosphorylation at residue D59 by an as-yet-unidentified histidine kinase [83]. CsgD directly activates transcription of the *csgBAC* operon by binding to a specific promoter motif [9]. In contrast to *E. coli*, *S.* Typhimurium CsgD does not positively autoregulate its own promoter [77,84]. Instead, its expression is controlled by multiple regulators belonging to diverse regulatory pathways [8,9,10]. Positive feedback regulation occurs indirectly through CsgD-dependent transcription of genes encoding AdrA that modulates cellulose expression and IraP that stabilizes sigma factor RpoS [17,84,85,86]. These regulators are also involved in curli production and will be discussed in subsequent sections. Beyond curli regulation and cellulose production, CsgD also upregulates genes encoding other essential biofilm matrix components, including BapA and the O-antigen capsule [14,87]. BapA plays a role in bacterial virulence and contributes to intestinal colonization [14]. The O-antigen capsule, encoded by the *yih* operons (*yihU-yshA* and *yihVW*), is an important component of the extracellular matrix [87].

The two *csg* operons are typically expressed toward the end of the exponential growth phase and during the stationary phase [88]. However, extensive bacterial growth leads to lower CsgD expression, thereby limiting de novo curli production once the biofilm has matured [89]. CsgD functions as a bistable regulator: even at peak expression levels, there are two distinct subpopulations of bacteria consisting of CsgD-producing (CsgD-ON) and non-producing (CsgD-OFF) cells [84,89,90].

In *S.* Typhi, the *csgD* has a nonsense SNP at its 3′ end, resulting in the truncation of the regulator by 8 amino acids at its carboxyl terminus [50,91]. This truncation prevents CsgD from binding to the *csgBAC* promoter region [27]. Given that CsgD is unable to bind the *csgB* promoter in *S.* Typhi, its role in regulating downstream targets such as *adrA* and *bapA* remains unclear. Nevertheless, residual activity through binding to higher-affinity sites cannot be excluded. Although cellulose production is thought to be impaired in *S.* Typhi [14,50], as *bcsC* is a pseudogene, a cellulose-positive phenotype was observed in some clinical isolates and by heterologously expressing *S.* Typhimurium CsgD [92]. These findings suggest that alternative regulatory pathways may compensate for the lack of CsgD activity in *S.* Typhi. Consistent with this hypothesis, correction of the *csgD* nonsense mutation did not restore curli expression to levels comparable to wild-type *S.* Typhimurium, suggesting that CsgD is not the central regulator of curli expression in *S.* Typhi [27,92]. Despite the impaired function of CsgD, transcription of the downstream genes of the operon (*csgE*, *csgF* and *csgG*), which are under control of the *csgD* promoter, could still occur.

## 7. Curli Regulation

### 7.1. Osmolarity

Curli expression is controlled by multiple regulatory pathways. Among them, the two-component systems (TCS) EnvZ/OmpR and CpxA/CpxR respond to osmotic, pH and envelope stress and have been identified as key regulators of curli production [93,94,95]. EnvZ is a transmembrane histidine kinase that modulates the intracellular level of phosphorylated OmpR (OmpR-P) through its dual kinase and phosphatase activities [96,97,98,99]. While the exact sensing mechanism is not well understood, accessory proteins such as the transmembrane protein MzrA can modulate EnvZ activity on OmpR [100]. In addition to EnvZ-dependent phosphorylation, OmpR can also be phosphorylated by cross-talk with other TCS or by acetyl phosphate (AcP) produced by the Pta/AckA pathway [101,102]. Phosphorylated OmpR binds to the promoter regions of target genes according to a hierarchy of high- and low-affinity binding sites, resulting in either activation or repression of expression [97,103,104,105]. The curli intergenic region contains six OmpR binding sites (D1, D2 and D3-D6) (Figure 2) [106]. The D1 site, located at −51 pb upstream of the *csgD* transcription site, has the highest affinity with OmpR-P and is the activating site. Under low osmolarity conditions, OmpR-P levels are limited, favoring preferential binding to D1. Unphosphorylated OmpR can also bind this site, with lower affinity [106]. At high osmolarity, increased OmpR-P levels promote binding to lower-affinity sites such as the inhibiting sites D2 (at −71 pb) and D3-D6 (at −86 to −267), resulting in repression of *csgD* expression [106]. Binding of OmpR to multiple sites promotes its dimerization and induces conformational changes in the DNA, thereby influencing transcriptional output [105,107,108]. In *S*. Typhimurium, OmpR is an essential activator of the *csgD* promoter, as a deletion of *ompR* drastically reduces *csgD* expression and attenuates virulence [26,106,109,110]. The SNPs in the *S.* Typhi intergenic region produce two mutations within the OmpR D1 binding site and one at the end of the D3-D6 sites (Figure 2A). These SNPs are highly conserved among *S.* Typhi serovar strains (Figure 2B). While the OmpR amino acid sequence is conserved between *S.* Typhimurium and *S.* Typhi, EnvZ in *S.* Typhi contains one missense mutation (Table 1). This substitution reduces the EnvZ autophosphorylation rate and consequently decreases OmpR phosphorylation [111]. This potentially alters the expression of the OmpR regulon [111]. A reduction in OmpR phosphorylation could lead to less efficient binding to the curli intergenic region and thus a shift in the binding hierarchy with competing proteins such as CpxR [26]. However, the specific impact on curli regulation remains to be fully elucidated.

In *E. coli*, the TCS CpxA/CpxR further inhibits curli production in addition to OmpR under high-osmolarity conditions [112]. CpxR binds directly to the curli intergenic region, adjacent to the OmpR D1 activating site, thereby interfering with OmpR-dependent activation [20,112]. Within the Cpx system, CpxA functions as the sensor histidine kinase, whereas CpxR acts as the response regulator [113]. The Cpx system responds primarily to membrane stress signals, including alkaline pH, iron availability, periplasmic protein misfolding, and overproduction of membrane proteins [114]. The latter is particularly relevant for curli regulation, as excessive production of curli subunits activates the Cpx pathway, which in turn represses curli expression [20]. Expression of *cpxR* is RpoS-dependent and upregulated during the stationary phase and under stress conditions [115].

While the direct effect of the Cpx system on *Salmonella* curli regulation is not well defined, two binding sites have been predicted on the intergenic region of curli in *Salmonella*: one on the promoter of *csgB* and one near the D1 binding site of OmpR [26]. In addition, expression of *cpxP*, a CpxA inhibitor, has been shown to be upregulated during biofilm formation in *S.* Typhimurium [116]. This suggests that the Cpx system could also be directly involved in curli regulation in *Salmonella*. In *S.* Typhi, the *cpxR* gene contains a missense SNP (Table 1). This substitution is unlikely to impair CpxR function, as it is a conservative mutation and does not affect the phosphorylation site (D51) nor the DNA-binding domain [117].

### 7.2. pH

The direct effect of pH on curli regulation is still unclear. However, the TCSs EnvZ/OmpR and CpxA/CpxR are both responsive to pH fluctuation [118,119]. During acid stress, EnvZ undergoes conformational changes upon periplasmic acidification, which in turn alters OmpR conformation and stimulates its DNA-binding capacity independently of phosphorylation [119]. In parallel, AcP can phosphorylate OmpR independently of EnvZ during the acid stress response [120], further modulating OmpR activity. OmpR also contributes to cytoplasmic acidification under acid stress conditions by repressing the lysine decarboxylation pathway [119,121]. Under high-osmolarity and low pH conditions, OmpR directly binds to and represses the general stress sigma factor RpoS, thereby influencing intracellular pH homeostasis, including regulation of genes such as *yghA* [120]. RpoS itself plays a central role in the acid tolerance response (ATR) [122]. Importantly, RpoS activates transcription of the *csgD* and *csgB* promoters, primarily at the transition from the exponential phase to the stationary phase [9,82,88,123]. While RpoS is important for curli expression, RpoD can also recognize the *csgB* promoter and activate expression in the absence of RpoS and H-NS [88].

The small RNAs ArcZ, RprA and DsrA activate RpoS expression during acid stress [124,125,126]. DsrA can also regulate H-NS by increasing mRNA turnover in response to acid stress [127,128]. H-NS is a histone-like protein that modulates DNA-structuring and gene regulation and binds preferentially to AT-rich regions with low specificity [129,130,131]. This potentially inhibits curli expression downstream, as H-NS could impede binding of RpoS even if it is highly expressed at acidic pH. Among the three sRNAs, RprA is the only one that contains a SNP in *S.* Typhi (Table 1). Conversely, the CpxA/CpxR system responds to alkaline stress. CpxR activation occurs via CpxA-dependent phosphorylation or through AcP, linking envelope stress and metabolic status to curli regulation [118].

Additional two-component systems contribute to the ATR, including the RcsCDB phosphorelay system and RstAB [132,133]. The Rcs system is activated under multiple stress conditions, such as acid stress, high osmolarity and desiccation [132,134,135,136]. It consists of the hybrid sensor kinase RcsC, the histidine phosphotransfer protein RcsD, and the response regulator RcsB. The outer membrane protein RcsF interacts with IgaA to activate the Rcs pathway, thereby enabling signal transduction [137,138]. Phosphorylation of RcsB can occur via RcsC or RcsD [139,140,141]. Different phosphorylation states of RcsB modulate its ability to form homo- or heterodimers with accessory proteins such as RcsA [139,140].

In *S.* Typhimurium, unphosphorylated RcsB is essential for upregulation of biofilm and curli expression [142]. In contrast, phosphorylated RcsB inhibits *csgD* expression indirectly by inducing transcription of the sRNA RprA [142,143]. RprA enhances RpoS translation while inhibiting *csg* expression [144,145]. Thus, under stress conditions such as ATR, increased RcsB phosphorylation inhibits curli production. Recently, a predicted binding site of RcsB was identified in the curli intergenic region of *E. coli*, overlapping with H-NS sites [146]. The binding site in the *Salmonella* sequence has yet to be confirmed.

In *S.* Typhi, while RcsF is conserved, multiple SNPs are present in other genes encoding components of the Rcs system. These include substitutions in the histidine kinase and response regulator binding domain of RcsC [147], and in the phosphotransfer (HPt) domain of RcsD [148]. Both RcsB and RcsA encoding genes also contain mostly conservative substitutions that are not within regions corresponding to predicted functional domains (Table 1). In addition, the *igaA* gene contains mutations encoding the periplasmic and cytoplasmic portions of the protein (Table 1). Although the functional consequences of these SNPs remain unclear, they may alter phosphoryl transfer efficiency and potentially impact downstream regulation of curli expression.

The RstAB TCS also contributes to acid stress adaptation and curli regulation and can cross-talk with the Rcs system [149]. RstB is the histidine sensor kinase and RstA is the response regulator. Expression of *rstBA* is controlled by the PhoPQ TCS [150], which responds to acidic pH and low Mg^2+^ conditions [151]. In contrast to *E. coli*, *rstA* and *rstB* are separated by additional genes in *Salmonella*. RstA directly activates *csgD* expression under acidic conditions and modulates RpoS stability, thereby influencing curli production [133,152]. This may ensure a basal level of *csgD* expression under favorable conditions. In *S.* Typhi, an SNP in *rstA* is found in the segment encoding the predicted regulatory domain of RstA, and multiple SNPs in *rstB* are present in regions encoding the histidine kinase domain, which could affect signaling efficiency (Table 1).

### 7.3. Temperature

Bacteria are subject to drastic temperature changes by transiting from the environment (below 25 °C) to their human host (37 °C). Curli expression is temperature-dependent. Curli is highly expressed at 30 °C and below in *Salmonella* and *E. coli* [25]. However, curli promoters are more expressed at 37 °C in *S.* Typhi [27]. This is not surprising, as *S.* Typhi is a human-specific serovar, and certain *E. coli* strains also produce curli and biofilm in human hosts, adhering to intestinal epithelial cells at physiological temperature in vitro [31,153,154]. Some pathogenic strains of *E. coli* also express curli at 37 °C [155].

The temperature regulation of curli is highly associated with the post-transcriptional regulation of the sigma factor RpoS [156]. Pseudo-chaperone Crl and small RNA DsrA favor RpoS translation and binding to the curli promoter region in a temperature-dependent way [157,158,159,160]. Crl activity is optimal at 30 °C and low at 37 °C, thus acting like a thermosensor [158,161]. Although Crl is required for the maximal expression of curli and cellulose in *S.* Typhimurium, Crl is only partially responsible for RpoS regulation of *csgD*. The depletion of *crl* does not abolish *csgD* expression [159,161]. At temperatures below 30 °C, the small RNA DsrA is increased and protects RpoS mRNA from degradation by increasing its stability [160,162]. Thus, DsrA also acts as a thermosensor for RpoS. However, it was previously reported that DsrA could have a lesser effect on RpoS in *Salmonella* compared to *E. coli* [144]. No SNPs are found in the genes encoding either DsrA or Crl in *S.* Typhi. This raises the question of how *S.* Typhi regulates curli in response to temperature. Certain *S.* Typhimurium strains carry mutations in the *csgD* promoter that cause constitutive expression of *csgD*, independent of temperature and independent of RpoS [25]. It was suggested that these mutations alter the specificity of the RNA polymerase (RNAP), which could load another sigma factor, such as RpoD [25].

H-NS is temperature-regulated [163,164]. Dimerization of H-NS promotes DNA binding to modulate gene expression, and oligomerization of H-NS promotes chromosome condensation [165,166,167,168]. H-NS dimers undergo conformational changes at 37 °C and have reduced DNA affinity, which relaxes DNA binding and enables expression of silenced genes [163]. H-NS activates and represses genes at lower temperatures (below 30 °C) and 37 °C [163,164,169]. H-NS binding sites are situated in the intergenic region of curli, across approximately 270 bp, from −69 to −339 bp upstream of *csgD* [106] (Figure 2). CsgD potentially acts as a counter-silencer by competing with H-NS sites. CsgD bends the DNA, which allows RpoS to initiate expression [123]. H-NS also regulates RpoS and DsrA expression levels and subsequently influences curli expression [164]. Accumulation of RpoS during the stationary phase contributes to the release of DNA from H-NS [170]. Four SNPs are found in the H-NS binding sites within the *csg* intergenic region of *S.* Typhi (Figure 2). H-NS does not have a conserved specific recognition binding site but binds preferentially to the AT-rich region [171]. Thus, the four SNPs scattered throughout its predicted site might not impact its regulation of curli expression in *S.* Typhi. Further investigation is required to determine the effect of those SNPs on the regulatory pathway.

RspA is a novel regulator that has a putative dehydratase function. It is known in *E. coli* to be involved in starvation-sensing [172]. Recently, RspA was identified as acting as a temperature sensor for biofilm components in *S.* Typhimurium. Cellulose and curli production were affected by RspA in a temperature-dependent manner [81]. Further studies are required to determine whether RspA directly regulates these components. RspA of *S.* Typhi contains a few amino acid substitutions (three non-conservative and two conservative changes) with one mutation present in the predicted active domain of the protein (Table 1).

MlrB (also known as ORF319) and BrfS are MlrA homologs specific to *Salmonella*. MlrB, encoded within SPI-2, represses *csgD* expression during intracellular infection, thereby limiting curli production inside host cells [173]. In *S*. Typhi, the *mlrB* gene contains a single SNP located at the 5′ end of the gene that is not predicted to encode any functional domain (Table 1). In contrast, the recently identified DNA-binding regulator BrfS induces *csgBA* expression under low-temperature (20 °C) and nutrient-limiting conditions. BrfS directly induces both the *csgBA* and *csgD* promoter expression, which promotes curli production, suggesting a role for environmental persistence at low temperatures. Meanwhile, it also represses flagellar gene expression [174,175]. In *S*. Typhi, the *brfS* gene contains two SNPs, one within the region encoding the DNA-binding domain and another located in a section encoding the C-terminal end of the protein (Table 1). The BrfS regulatory system is likely non-functional in *S.* Typhi because the bacterium has evolved into a human-restricted pathogen that no longer needs to survive in cold, external environments. However, the functional impact of these polymorphisms remains to be determined, but they may influence regulator activity and, consequently, curli expression.

### 7.4. Oxygen Tension

The ArcBA TCS is a key regulator of anaerobic respiration and has been shown to influence *csgD* expression. Under oxygen-limiting conditions, the histidine kinase ArcB senses the reduction of quinones to quinols, leading to its autophosphorylation and subsequent activation of the response regulator ArcA [176,177,178,179]. In contrast, during aerobic respiration, quinones remain oxidized, preventing activation of the ArcBA system [177,180]. Although ArcBA is primarily associated with oxygen sensing, carbon source availability also modulates its activity independently of oxygen levels [177]. Quinones are reduced in nutrient-rich and exponential growth conditions, whereas they remain oxidized during starvation or the stationary phase [181,182]. There might be a link between oxygen tension and the osmolarity regulation pathways mediating curli expression. In *E. coli*, ArcB can also activate OmpR through non-cognate phosphorylation under anaerobic conditions [183]. For curli regulation, phosphorylated ArcA represses *csgD* expression both directly and indirectly. It inhibits *rpoS* transcription and represses the sRNA ArcZ, a positive regulator of RpoS translation that directly upregulates *csgD* expression [125,177,184,185]. In addition, ArcA directly binds to the *csgD* promoter region to repress *csgDEFG* transcription [186,187]. ArcB indirectly contributes to RpoS regulation by phosphorylating RssB, which targets RpoS for degradation via the ClpXP protease under anaerobic or nutrient-rich conditions [177,188,189]. Altogether, activation of the ArcBA system leads to decreased RpoS levels and inhibition of *csgD* expression, thereby limiting curli production under anaerobic and high-carbon conditions [24,27]. In *S.* Typhi, ArcB contains three conservative amino acid changes in the histidine kinase domain, one in the response regulator interaction domain, and two in the HPt domain, whereas ArcA is fully conserved. The functional consequences of these changes remain to be determined, but they may influence signal transduction efficiency and downstream regulation.

The integration host factor (IHF) is a nucleoid-associated protein composed of α- (IhfA) and β- (IhfB) subunits that form a heterodimer that is highly abundant in bacterial cells. IHF binds specific DNA sequences within the minor groove and induces a pronounced bend of approximately 160°, thereby influencing transcriptional regulation [24,190,191,192]. While IHF is not directly linked to oxygen sensing, curli expression in an *ihf* mutant has been shown to depend on oxygen availability [26,106]. In *S.* Typhimurium, an *ihf* mutant displays a reduced rdar phenotype compared to the wild-type strain under microaerophilic growth, while no significant difference is observed under aerobic growth [26,106]. This suggests that IHF contributes to oxygen-dependent regulation of curli expression. Predicted IHF binding sites have been identified in the *csgD* promoter region, one upstream at −181 pb and another within the coding sequence at +273 pb in *S.* Typhimurium [106]. Notably, the upstream site overlaps with the OmpR binding inhibitory region (D3-D6), suggesting competition between IHF and OmpR. In this context, IHF binding may impede OmpR-mediated repression, thereby promoting *csgD* expression [26]. In addition, a study in *Legionella pneumophila* showed that RpoS can directly activate *ifhAB* expression [193]. Although this regulatory relationship has not been confirmed in *Salmonella*, it raises the possibility that IHF could indirectly respond to oxygen availability through RpoS-dependent pathways.

Oxygen tension does not influence *csg* expression in *S.* Typhi in the same way as in *S.* Typhimurium. While *csgB* promoter activity in *S.* Typhi is generally higher under aerobic conditions, temperature remains the dominant factor, with maximal expression observed at 37 °C independently of oxygen levels [27]. In contrast, in *S.* Typhimurium, *csg* expression peaks at 30 °C and is strongly dependent on aerobic conditions [27]. These observations suggest that oxygen tension plays a more limited role than temperature in regulating curli expression in *S.* Typhi. This distinction further highlights the regulatory divergence between both serovars. Curli expression is governed by the combined effects of multiple environmental cues, emphasizing the importance of signal integration in fine-tuning biofilm formation.

### 7.5. c-di-GMP

The second messenger cyclic di-GMP (c-di-GMP) plays a critical role in the transition between motile and sessile lifestyles, as well as in the cell cycle, differentiation, virulence, and biofilm formation, with the latter being the most extensively studied [85,194,195]. c-di-GMP is synthesized by diguanylate cyclases (DGCs) containing GGDEF domains and degraded by phosphodiesterases (PDEs) containing EAL or HD-GYP domains [196,197,198,199].

In *S*. Typhimurium, 19 predicted enzymes involved in c-di-GMP metabolism have been predicted, including 5 proteins containing GGDEF domains, 7 with EAL domains, and 7 with dual GGDEF/EAL domains [200]. Although not all of them directly regulate curli expression, many of these enzymes contribute to processes such as epithelial cell invasion, host inflammatory responses, and intestinal colonization [86,201]. Elevated intracellular c-di-GMP levels promote the production of extracellular matrix components, such as cellulose and curli fimbriae, while decreasing bacterial flagella-mediated motility [86,202,203]. Conversely, host-derived nitrate produced during inflammation decreases c-di-GMP levels, thereby repressing curli expression [41]. Similarly, L-arabinose also inhibits *csgD* expression via modulation of c-di-GMP pools in *Salmonella* [204].

A key regulatory loop influencing curli expression involves the diguanylate cyclase AdrA, which is positively regulated by CsgD. In turn, c-di-GMP produced by AdrA promotes *csgD* transcription and activity, forming a positive feedback loop [86]. However, AdrA accounts for only a fraction of the total c-di-GMP-mediated regulation of curli [86]. In *S.* Typhi, the *adrA* gene contains multiple SNPs scattered throughout the gene, resulting in changes to the integral membrane sensor domain (MASE2) (two SNPs), the GGDEF domain (one SNP) and two more SNPs in non-characterized regions, and these may affect its regulatory activity.

Additional DGC enzymes, such as STM3388, STM2123 (YegE), STM4551 and STM1987 (YedQ), act as *csgD* activators, whereas PDEs such as STM1703 (YciR), STM4264 (YjcC), STM3611 (YhjH) and STM1827 function as repressors [86,202]. STM1703 is bifunctional and contains both GGDEF and EAL domains. Two additional EAL-containing proteins, STM1697 and STM1344 (YdiV), lack catalytic activity but indirectly enhance curli expression by targeting the flagellar regulator Flh, thereby relieving inhibition mediated by YciR and YhjH [205,206]. Specific DGCs such as STM3388 and YegE contribute to ~60% of *csgD* regulation without affecting global c-di-GMP levels, demonstrating the importance of localized cellular c-di-GMP signaling pools contributing to the regulation of biofilm formation, curli, and flagella-mediated motility [86]. Mutations in some DGCs reduce *csgD* expression and result in a smooth and white phenotype on Congo red agar [207]. The PDE YciR may act locally at the *csgD* promoter, in addition to its role in global c-di-GMP regulation. In addition, it contributes to the rdar phenotype [207]. The DGC YegE is expressed early during growth, and its deletion reduces *csgD* expression at early stages [86]. In the stationary phase, RpoS induces the expression of YegE and YedQ, leading to activation of the effector YcgR, which inhibits flagellar motility and suppresses YhjH activity, thereby promoting curli production [208]. Overall, increased RpoS levels favor c-di-GMP accumulation and curli expression across multiple growth conditions. In *S.* Typhi, multiple polymorphisms are present across DGC and PDE enzymes, including amino acid substitutions within catalytic domains (Table 1). Moreover, the EAL domain of STM3388 (STY3568) is truncated by 174 amino acids out of 639 in *S.* Typhi, suggesting potential loss of function. In addition, the EAL domain of STM1697 (STY1354) of *S.* Typhi is predicted to be 57 amino acids longer than that of *S.* Typhimurium (Table 1). In *E. coli*, deletion of c-di-GMP turnover proteins such as YciR, YedQ and AdrA leads to temperature-independent CsgD regulation [209,210,211]. In *S.* Typhimurium, deletion of YjcC also causes temperature-independent regulation of CsgD [202]. These enzymes and all the other discussed DGEs and PDEs harbor multiple substitutions in their GGDEF, EAL or sensor domains. This could contribute to *S.* Typhi responding differently to temperature signals than *S.* Typhimurium, with preferentially higher *csg* expression at 37 °C rather than 30 °C [27].

Additional regulatory systems contribute to the modulation of intracellular c-di-GMP pools and, consequently, curli expression. The PhoPQ TCS enhances biofilm formation and curli production in part via induction of c-di-GMP production [212]. The DsbBA system plays a key role in oxidative stress response and protein folding. DsbA is a periplasmic disulfide oxidase, while DsbB is a membrane-bound oxidoreductase responsible for stabilizing protein disulfide bonds [213,214]. Under oxidative stress, activation of DsbA can influence other regulatory pathways, including RcsCDB [132], PhoPQ [215] and CpxAR [20] regulons, collectively leading to repression of curli expression [216]. The DsbBA system inhibits biofilm formation on solid surfaces but is required for pellicle formation, suggesting a role in oxygen-dependent biofilm regulation [20,132,215]. Deletion of either *dsbA* or *dsbB* increases curli expression through modulation of YdiV and STM3615 (YhjK) [216]. In *S.* Typhi, PhoP and PhoQ each carry modifications on functional domains. DsbA contains two changes in the thioredoxin domain, whereas the DsbB protein remains fully conserved (Table 1).

The BarA/SirA TCS regulates virulence, metabolism, and motility in *S.* Typhimurium [217,218,219,220]. The sensor histidine kinase BarA responds to environmental cues such as pH, bile, and short-chain fatty acids, and phosphorylates the response regulator SirA [221,222,223], which can also be activated by AcP [223]. SirA induces expression of the sRNAs CsrB and CsrC, which sequester the global regulator CsrA. CsrA modulates c-di-GMP pools by regulating multiple DGCs and PDEs [224]. CsrA also promotes motility by activating flagellar genes and by inhibiting YdiV [220], while generally exerting a negative effect on curli production by downregulating DGCs (e.g., YedQ, STM4551) and upregulating PDEs such as YhjH [10]. However, CsrA also downregulates some PDEs (YciR and STM1827) [10], highlighting its complex and context-dependent regulatory role. In addition, overexpression of CsrB and CsrC increases biofilm formation and curli expression [220,225], suggesting an overall inhibitory role of CsrA on curli production. The BarA/SirA system also interacts with the RcsCDB regulon, promoting RcsB activation during exponential growth in rich media via CsrB/CsrC-mediated regulation [226]. In *S*. Typhi, BarA contains three conservative amino acid substitutions, while SirA is fully conserved. CrsB contains four amino acid changes dispersed across the sequence, while CrsC and CrsA are fully conserved (Table 1).

The transcription factor MlrA (formerly YehV), a member of the MerR family, is another key regulator of *csgD* [227]. Its expression is activated by RpoS and therefore increases during the stationary phase. MlrA activity is also positively influenced by elevated c-di-GMP levels, linking environmental signaling to transcriptional control [24,153]. MlrA is essential for curli production, as its deletion abolishes *csgD* expression [227,228]. Its predicted binding site is located upstream of the OmpR D2 site and overlaps with H-NS binding regions, suggesting coordinated or competitive interactions among these regulators [229]. MlrA may also interact with the RpoS-dependent transcriptional machinery, which activates *csgD* expression [227]. In *E. coli*, MlrA regulates two important DGCs, YegE and YdaM (also known as DgcM), that are responsible for *csgD* expression [227]. While the YegE homolog can be found in *Salmonella*, YdaM is absent [195]. Thus, regulation of curli expression by the c-di-GMP pool is different in *Salmonella*. In *S*. Typhi, both conservative and non-conservative SNPs are present within the *mlrA* gene, corresponding to changes within the C-terminal region of the protein (Table 1).

Evidence has shown that the second messenger c-di-GMP can directly interact with H-NS, promoting its dissociation from DNA at high intracellular concentrations [230]. This mechanism may relieve H-NS-mediated repression of *csgD*, further supporting the link between elevated c-di-GMP levels and enhanced curli expression. However, further investigations are required.

Overall, c-di-GMP signaling represents a highly integrated regulatory network, modulated by multiple environmental cues and regulatory pathways. The interplay between global regulators, small RNAs, and enzymatic control of c-di-GMP pools ultimately determines the level of *csgD* expression and curli production.

### 7.6. Other Small RNAs

Small regulatory RNAs (sRNAs) are non-coding RNAs that modulate gene expression via antisense base-pairing with the target sequence, thereby affecting gene expression and translation [231,232]. In the context of curli regulation, most sRNA-mediated interactions occur within the 5′ untranslated region (UTR) of *csgD*, which serves as a major regulatory hotspot [232]. Several key sRNAs, including RprA, DsrA, ArcZ, GcvB and CsrB/CsrC, have been discussed in previous sections. Additional sRNAs such as SdsR, RydC, OmrA/OmrB and GcvB also contribute to curli regulation in *Salmonella* [185,233]. McaS, which inhibits *csgD* expression while promoting motility and regulating carbon metabolism through CRP, is absent in *Salmonella* [234]. Other sRNAs affecting curli expression were reported in *E. coli* [225]; however, their roles in *Salmonella* remain to be fully characterized.

SdsR is highly expressed during the stationary phase and regulates a broad range of targets, including membrane proteins, metabolic enzymes and DNA-binding proteins [235,236]. It has been reported to positively regulate *csgD* expression, although no direct binding site has been identified, suggesting an indirect mechanism [185]. SdsR negatively regulates RpoS but requires RpoS for its own transcription, forming a negative feedback loop [185,237]. Thus, SdsR regulation of *csgD* may be indirect due to its regulation of RpoS.

RydC is involved in the regulation of the *yej* operon, which contributes to virulence, antimicrobial resistance, and intracellular survival [238]. It also activates cyclopropane fatty acid (CFA) synthase, thereby influencing membrane stability [239]. RydC reduces curli and biofilm production by forming a pseudoknot structure with *csgD* mRNA, a process facilitated by the RNA chaperone Hfq [233]. This interaction alters mRNA conformation by exposing the 5′ domain and promotes translational repression.

OmrA and OmrB, which are regulated by OmpR [240], contribute to iron homeostasis by inhibiting the expression of enterobactin receptors CirA and FepA [240]. Since curli expression is enhanced under low-iron conditions [24,25], these sRNAs indirectly influence curli production and even bypass temperature regulation [241]. OmrA/OmrB also directly inhibit *ompR* and *csgD* by binding to their UTRs, creating a regulatory feedback loop [240,242].

The sRNA GcvB plays a role in acid stress response, amino acid metabolism, and RpoS-dependent regulation [243,244]. GcvB is regulated by GcvA and is induced in the presence of glycine [245]. It is highly expressed in acidic, nutrient-rich conditions and during exponential growth [246,247]. In *E. coli*, under these conditions, GcvB directly represses *csgD* expression by binding to a site overlapping the RprA binding region [248,249,250]. While the effect of GcvB on *Salmonella* curli expression is unknown, the GcvB regulon is highly conserved in *Enterobacteriaceae* [251].

Most sRNAs require the RNA chaperone Hfq to stabilize their structure and facilitate interactions with target mRNAs [252]. Hfq stabilizes sRNAs by preventing degradation and enhances their regulatory efficiency [233]. In *S.* Typhimurium, Hfq influences the expression of approximately 20% of genes [253]. Deletion of *hfq* results in loss of RpoS production and, consequently, reduced *csgD* expression. However, complementation with RpoS alone does not restore curli production, indicating that Hfq also directly regulates *csgD* independently of RpoS [185,254]. Overall, sRNAs, together with Hfq, form a complex post-transcriptional regulatory network that fine-tunes *csgD* expression in response to environmental and cellular cues.

### 7.7. Other Regulators

Curli regulation is mediated by a wide array of additional factors that integrate environmental and cellular stimuli. Housekeeping transcription factors such as GreA and GreB contribute to efficient *csgD* transcription elongation within the 5′ UTR, thereby influencing curli expression [255]. Beyond their general roles in transcriptional fidelity, these ubiquitous factors are also involved in transcription initiation by releasing stalled polymerases and refolding misfolded proteins [256,257,258]. In *Salmonella*, they are also involved in virulence-associated pathways [259]. In *S.* Typhi, a SNP resulting in a change in the C-terminal RNA polymerase binding site of GreB may affect its regulatory efficiency, whereas GreA is fully conserved (Table 1).

Oxidative stress further modulates curli expression through the SoxRS system. At 37 °C, SoxRS inhibits *csgD* transcription by directly binding upstream of its promoter and interfering with other regulators [260]. SoxR is the intracellular sensor that detects redox signals that activate SoxS expression. In contrast, oxidative stress at lower temperatures (<30 °C) promotes curli and biofilm formation [241,261], highlighting the importance of environmental cues. In *S.* Typhi, SoxS contains a conservative amino acid substitution in the DNA-binding domain, whereas SoxR is fully conserved (Table 1).

Several other regulators identified in *E. coli*, such as the Tol-pal system [134,262], YhjC [263], YiaJ [263], BolA [264], SdiA [265], MqsA [266], and Fis [154] have been implicated in curli regulation, but their potential roles for regulation of curli or biofilm production in *Salmonella* remain largely unexplored.

## 8. Conclusions

Curli fimbriae are important determinants of biofilm formation and environmental persistence in several bacteria, including *Salmonella enterica* serovar Typhimurium (*S.* Typhimurium) [8,9,10]. For *S.* Typhi, persistence is rather associated with its human host [7]. Thus, curli regulatory pathways in *S.* Typhi are likely adapted to host-associated rather than environmental signals. In contrast to *S.* Typhimurium, *S.* Typhi does not induce strong intestinal inflammation at the early stage of infection [43,267,268]. Curli fibers were shown to activate the inflammatory response through Toll-like receptor 2 (TLR2) [269,270]. Thus, repression of curli production may contribute to immune evasion and systemic dissemination in *S.* Typhi. This could explain the dysregulation of *S.* Typhi curli in comparison to *S.* Typhimurium. Although curli expression may be suppressed during acute infection, curli could still be produced during later stages of the infection cycle, particularly during persistence. *S.* Typhi produces biofilm in the gallbladder of 3–5% of infected patients, representing the only known reservoir of the bacterium [5]. Human bile was shown to upregulate *csgA* expression in *Salmonella* biofilms [271].

**Figure 3:**
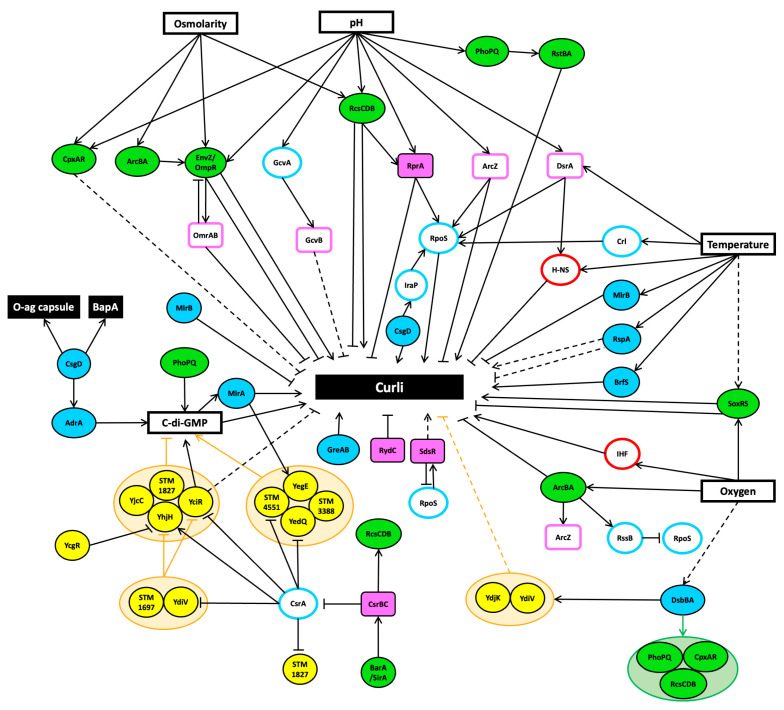
Schematic representation of the curli fimbriae regulation pathway in *S*. Typhimurium. The curli genes and intergenic region are represented in a single black box (curli) at the center of the figure. Other products (BapA and O-ag capsule) are also in a black box. Environmental cues (i.e., osmolarity, pH, temperature and oxygen) are in white boxes. Every element of the regulation pathway is featured as follows: TCS (green circles), other regulators (blue circles), nucleotide-structuring proteins (red circles), DGC and PDE (yellow circles) and sRNAs (pink rectangles). The relationship between each element is represented by sharp head arrows (activation), blunt head arrows (inhibition), and full (direct regulation) and dashed (prediction) lines. Elements harboring SNPs are shown as fully colored, while elements that are 100% homologous are represented with a white center and a colored outline. Regulators sharing similar regulatory effects are grouped within a larger circle connected by a colored arrow. When the regulation arrows point to the large circle, the regulation applies to all the elements inside the circle, whereas when the arrows single out one element inside the circle, the regulation only applies to that element.

Curli fibers are composed of the structural subunits CsgA and CsgB, which are exported to the bacterial surface with the export machinery composed of CsgE, CsgF and CsgG secretion machinery [8]. In *E. coli* and in non-typhoidal *Salmonella* serovars such as *S.* Typhimurium, the transcriptional regulator CsgD activates expression of the *csgBAC* operon [82,123]. However, several polymorphisms are present within *S.* Typhi *csg* genes. The most critical difference resides in a nonsense mutation in *csgD* that prevents CsgD from binding the *csgB* promoter [27]. Although this mutation likely disrupts canonical CsgD-mediated regulation, alternative regulatory pathways may compensate for the loss of CsgD function in *S.* Typhi, since the addition of the *csgD* allele from *S.* Typhimurium does not restore the rdar phenotype [27]. Even in *S.* Typhimurium, curli is regulated by a complex and dynamic network that integrates multiple environmental signals (Figure 3). Several regulatory systems are involved in multiple stress–response pathways, for example, EnvZ/OmpR and CpxA/CpxR respond to both osmolarity and pH change [93,94,95]. Rather than responding to individual cues, curli expression results from the combined effects of multiple physical and chemical conditions, including temperature, osmolarity, pH, oxygen availability, and intracellular c-di-GMP levels (Figure 3). Most studies have focused on a single growth condition, providing only partial insight into the regulatory pathways involved.

In *S.* Typhi, many components involved in curli regulation harbor mutations (Table 1). All the listed two-component systems, diguanylate cyclases and phosphodiesterases contain polymorphisms, including substitutions, single-nucleotide deletions, truncations and insertions. In silico analyses predict that several of these mutations affect conserved or functional protein domains, such as enzyme active sites, DNA-binding domains, and sensor regions. In addition, sequence polymorphisms are present in regulatory sRNAs, including RprA, GcvB and CsrB (Table 1). These modifications could alter curli regulation in *S.* Typhi by affecting signal sensing, protein–protein interactions, and protein–DNA interactions. Functional complementation experiments using *S.* Typhimurium alleles could help determine the contribution of these mutations to curli regulation in *S.* Typhi.

The *csg* intergenic region represents an additional level of regulatory complexity. This region harbors multiple overlapping transcription factor binding sites where numerous regulators compete for promoter occupancy and where regulatory outcomes depend on their relative abundance, binding affinity, activation state, and growth conditions. In *S.* Typhi, six SNPs are present within the *csg* intergenic region. These polymorphisms occur within predicted binding sites corresponding to the activating OmpR site D1, the inhibitory OmpR site D3-D6, and H-NS binding regions (Figure 2A). Such mutations could alter the binding affinity of OmpR and H-NS, thereby modifying *csg* gene expression. In addition, impaired binding of specific regulators under certain conditions may allow alternative regulators to access these sites and reshape the regulatory landscape. Correcting these SNPs in the *S.* Typhi *csg* intergenic region and assessing their effects on regulator binding and *csg* expression could provide important insights into the unique dynamics of curli regulation in *S.* Typhi.

Although a regulatory hierarchy model has been proposed in *E. coli*, the continuous identification of new regulators, genomic differences in bacteria, and the influence of different physical or chemical cues on curli expression highlight that regulatory mechanisms mediating curli regulation are far from fully resolved. While *S.* Typhimurium has served as the primary model to dissect these pathways, its regulatory framework cannot be directly extrapolated to *S.* Typhi. In *S.* Typhi, curli regulation remains poorly understood, and novel regulators could be identified through screening of transposon mutant libraries combined with analysis of *csg* gene expression.

These divergences point to a substantially rewired regulatory network. A deeper understanding of curli regulation in *S.* Typhi will require integrative approaches that account for combinatorial environmental signals and regulator interplay. Such efforts are essential to elucidate its biofilm biology and its role in persistence and pathogenesis.

## Figures and Tables

**Table 1:** In silico sequence comparison of members of the curli regulatory pathway between *Salmonella enterica* serovar Typhi and *Salmonella enterica* serovar Typhimurium.

Name	*S*Tm ^1^	*S*Ty ^2^	Role or Product	Modification in *S*Ty	Affected Predicted Domain ^3^
**Curli system components**					
CsgB	STM1143	STY1180	CsgA nuclease	G76E	R2
CsgA	STM1144	STY1181	Curli major subunit	-	-
CsgC	STM1145	STY1182	Anti-fibrillation protein	Q40L	Structure
CsgE	STM1141	STY1178	Accessory protein	Q36H	Self-association
CsgF	STM1140	STY1177	Accessory protein	-	-
CsgG	STM1139	STY1176	Porin-like protein	-	-
**Transcription Factors**					
CsgD	STM1142	STY1179	Biofilm master regulator	W209X	DNA-binding domain
OmpR	STM3502	STY3407	Osmolarity and pH regulator	-	-
CpxR	STM4059	STY3812	Osmolarity and pH regulator	S82N	REC domain
RpoS	STM2924	STY3049	RNA polymerase sigma factor 38	-	-
RcsB	STM2270	STY2495	Envelope stress and virulence regulator	V6I	Structure
RcsA	STM1982	STY2190	Transcriptional regulatory protein of RcsB	Q99K V204A	Structure Structure
RstA	STM1475	STY1647	Acid stress-response regulator	T43I	Structure
GcvA	STM2982	STY3122	Glycine cleavage system regulator	-	-
RspA	STM1505	STY1556	Temperature and starvation regulator	P42S R61H A247E L288I D377N	Structure Structure MR and MLE domain Structure Structure
MlrA	STM2160	STY2390	HTH transcription regulator	N219H R236H	C-terminal coactivator domain
MlrB	STM1389	STY1731	HTH transcription regulator	T17A	Structure
BrfS	STM1266	STY1855	HTH transcription regulator	R124W	DNA-binding domain
ArcA	STM4598	STY4947	Aerobic respiration control regulator	-	-
RssB	STM1753	STY1297	Adaptor protein of RpoS	-	-
PhoP	STM1231	STY1271	Environmental stress and virulence regulator	P130S	DNA-binding domain
BarA	STM2958	STY3096	Invasion regulator	D400E V542I V564I	Histidine kinase domain Structure Structure
GreA	STM3299	STY3477	Transcription elongation factor	-	-
GreB	STM3503	STY4293	Transcription elongation factor	R100T	RNA polymerase binding site
SoxS	STM4265	STY4463	Redox stress regulator	D19N	DNA-binding domain
**Sensors**					
EnvZ	STM3501	STY4295	Histidine kinase of OmpR	G354C	Autophosphorylation
MzrA	STY4295	STY3407	Modulator of EnvZ/OmpR	-	-
CpxA	STM4058	STY3813	Histidine kinase of CpxR	-	-
RcsC	STM2271	STY2496	Histidine kinase of RcsB	E505D V931A	Histidine kinase domain Response regulator binding domain
RcsD	STM2269	STY2494	Phosphotransferase of RcsB	A257T E860V D880V	Structure HPt HPt
RstB	STM1471	STY1651	Histidine kinase of RstA	E279K L287F T297N V305N H336R E409D	Histidine kinase domain Histidine kinase domain Histidine kinase domain Histidine kinase domain Histidine kinase domain Histidine kinase domain
ArcB	STM3328	STY3507	Histidine kinase of ArcA	I356V N460H V566A P694L A751V	Histidine kinase domain Histidine kinase domain Response regulator binding domain HPt HPt
PhoQ	STM1230	STY1270	Histidine kinase of PhoP	A168T	Sensor domain
SirA	STM1947	STY2155	Histidine kinase of BarA	-	-
SoxR	STM4266	STY4464	Histidine kinase of SoxS	-	-
**Nucleoid-associated proteins**					
H-NS	STM1751	STY1299	Histone-like nucleoid-structuring protein	-	-
IHFa	STM1339	STY1771	Integration host factor subunit alpha	-	-
IHFb	STM0982	STY0982	Integration host factor subunit beta	-	-
**c-di-GMP pathway**					
AdrA	STM0385	STY0418	DGE	P26S F52L V61S A160T V262A	Structure MASE2 domain MASE2 domain Structure GGDEF domain
-	STM3388	STY3568	DGE	A57E H165N N290S T305A D519A N526X	MHYT domain MHYT domain Structure GGDEF domain EAL domain Truncated EAL domain
YegE	STM2123	STY2336	DGE	K403Q T486A D498N D510N I676V H757L H819R L911P	PAC domain PAS domain Structure Structure GGDEF domain EAL domain EAL domain EAL domain
-	STM4551	STY4904	DGE	N25S V52G H85R T151A	Structure Structure Structure Structure
YedQ	STM1987	STY2194	DGE	L160del A161del R162del A171T S217P T237A R265Q A299N I340V L394P	Periplasmic sensor domain Periplasmic sensor domain Periplasmic sensor domain Periplasmic sensor domain Periplasmic sensor domain Structure Structure Structure Structure Structure
YciR	STM1703	STY1349	PDE	S100A A319V V597A	Structure GGDEF domain EAL domain
YjcC	STM4264	STY4462	PDE	I23V R201Q T215A I257V A363S I439V A514V	Structure CSS motif-EAL domain CSS motif-EAL domain EAL domain EAL domain EALdomain EAL domain
YhjH	STM3611	STY4192	PDE	A46T V52A H99R R116Q G153V T199I	EAL domain EAL domain EAL domain EAL domain EAL domain EAL domain
-	STM1827	STY1957	PDE	S32N V130A T173A A324T S443F A467V	Structure CSS motif-EAL domain CSS motif-EAL domain EAL domain EAL domain EAL domain
-	STM1697	STY1354	EAL containing protein	V162I R179ins *	EAL domain EAL domain elongation
YdiV	STM1344	STY1766	EAL containing protein	L110F L112V M140I	EAL domain EAL domain EAL domain
YhjK	STM3615	STY4188	GGDEF-EAL domain protein	M10V L153S Y466H N614D	Structure Structure EAL domain EAL domain
YcgR	STM1798	STY1926	Flagellar break protein	D47A E61K Q178P F182L I189L S190A E234D	Structure Structure PilZ domain PilZ domain PilZ domain PilZ domain Structure
CsrA	STM2826	STY2947	Carbon storage regulator	-	-
**sRNA**					
ArcZ	-	-	RpoS associated sRNA	-	-
RprA	-	-	RpoS associated sRNA	108A>U	-
DsrA	-	-	RpoS associated sRNA	-	-
GcvB	-	-	Acid biosynthetic related	-	-
CsrB	-	-	Starvation related sRNA	164G>A 172G>A 191A>T 225C>T	- - - -
CsrC	-	-	Starvation related sRNA	-	-
SdsR	-	-	*tolC* and *mutS* expression repressor	-	-
RydC	-	-	Regulator of *yejABEF*-encoded ABC permease	-	-
OmrA	-	-	OmpR-regulated sRNA	-	-
OmrB	-	-	OmpR-regulated sRNA	-	-
**Others**					
CsgI	STM1077	STY1099	Anti-fibrillation protein	-	-
HiuH (YedX)	STM1097	STY1129	5-hydroxyisourate hydrolase	D19N N25S	Structure Structure
IraP	STM0383	STY0415	Anti-adaptor protein of RpoS	-	-
Crl	STM0319	STY0364	Sigma factor-binding protein of RpoS	-	-
RcsF	STM0244	STY0271	Outermembrane lipoprotein	-	-
IgaA	STM3495	STY4301	Intracellular growth attenuator protein	I34F T642A	Cytoplasmic Periplasmic
DsbA	STM3997	STY3372	Periplasmic protein disulfide isomerase	V61M L90F	Thioredoxin domain Thioredoxin domain
DsbB	STM1807	STY1936	Putative disulfide oxidoreductase	-	-
Hfq	STM4361	STY4718	RNA-binding protein	-	-

-: Not applicable. * ins: insertion sequence in *S.* Typhi of YCDKIVVGGQENTRYLPALK TAGIWATQGTLFPSVALEEVETLLLGRRVNTLRESN. ^1^ Sequence of *Salmonella enterica* serovar Typhi CT18. ^2^ Sequence of *Salmonella enterica* serovar Typhimurium LT2. ^3^ Affected domains were determined by using in silico prediction tools and automatic annotation feature of Uniprot. When mutations are not in any predicted active domain, “structure” was used.

## Data Availability

No new data were created or analyzed in this study. Data sharing is not applicable to this article.
